# Supplementary material for: Non-Fatal Drowning Risk Prediction Based on Stacking Ensemble Algorithm
Source: Children (Basel). 2022 Sep 14;9(9):1383. doi: 10.3390/children9091383 (PMC9498184; doi:10.3390/children9091383)
Supplement: Supplementary file 1 [file children-09-01383-s001.zip › children-1842380-supplementary.pdf]

We have uploaded my **survey questionnaire (in English)** and **Table S1** as supplementary file. This will help the reviewers to understand the study more quickly.

Child Drowning Survey

Basic information

1. Your date of birth: YY/MM Age: Class: Year Class

2. Your gender ① Male ② Female

3. You consider yourself to be

①Introvert - quiet, not talkative, shy, like to do things alone

②Extrovert - lively, cheerful, active and like to talk to people

③Between introvert and extrovert - between active and quiet

④ Don't know

4. How do you think of your own curiosity?

① Very strong ② Strong ③ Not strong ④ Not strong at all

5. How is your relationship with your classmates?

① Very well ② Somewhat well ③ Not well ④ Not well at all

6. How is your relationship with your family?

①Very well ②Somewhat well ③Not well ④Not well at all

7. How many siblings are in your family (including yourself)?

① 1 ② 2 ③ 3 ④ 4 or more

8. What is your family ranking?

Drowning-related knowledge and behaviour

1. "Drowning is the leading cause of injury deaths among children and youth", is this correct?

① Correct ② Incorrect ③ Don't know

2. Do you think drowning among children and youth can be prevented?

① Yes ② No

3. Which of the following diseases do you think makes drowning easier when swimming?

① Heart disease ② Skin disease ③ Otitis media ④ Myopia

4. What is the best way to escape if the car you are in falls into the water?

① Turn on all the lights and wait for help.

② Break the windows and get out immediately.

③ Wait until the pressure inside and outside the windows is approximately equal, then open the windows and doors to escape.

④ Don't know what to do

5. Two minutes after drowning, people will be unconscious. How many minutes does the nervous system suffer irreversible damage?

① 4 to 6 ② 7 to 10 ③ 11 to 15 ④ Don't know

6. What do you think is the main function of inflatable swimming rings?

① Life-saving ② Play ③ Learn to swim ④ Don't know

7. What is the ratio of the frequency of cardiac compressions to the number of artificial breaths during CPR in children?① 5:1 ② 15:2 ③ 30:2 ④ Don't know

8. Would you still play or swim at the edge of a pond (or in a river, lake, reservoir, etc.) if there was a danger warning sign?

① Definitely ② Probably ③ Probably not ④ Definitely not

9. Is open water near your home or school well protected, e.g.

a. Are all wells covered? ① Yes ② No ③ No wells

b. Are all gutters fenced/covered? ① Yes ② No ③ No guttering

c. Are all construction site pools emptied in a timely manner? ① Yes ② No ③ No pools

d. Are all toilets/culvert pits fenced/covered? ① Yes ② No ③ No cesspit

e. Are all ponds, rivers, pools, etc. around your home marked with no swimming signs? ① Yes ② No ③ No streams, ponds, etc.

10. How far is your school surrounded by open water (e.g. ponds, rivers, reservoirs)?

① Within 100m ② 101-500m ③ Over 500m ④ None ⑤ Don't know

11. How far is open water (e.g. ponds, rivers, reservoirs) around your home?

① Within 100m ② 101-500m ③ More than 500m ④ None ⑤ Don't know

12. Is there any open water (e.g. ponds, rivers, reservoirs) on your way to school?

① Yes ② No ③ Don't know

13. If a friend or classmate asks you to go to a pond (or river, lake, reservoir, etc.) to play or swim without an adult, would you go?

① Definitely ② Probably ③ Probably not ④ Definitely not

14. How good are you at swimming?

① Proficient (swimming more than 100m) ② Average (swimming 50-100m)

③ Poor, can't swim 50m ④ Can't swim

15. In the past 12 months, have you swum in open water (ponds, rivers, lakes, reservoirs, etc.) unaccompanied by an adult?

① 3 times or more/month ② 1 to 2 times/month ③ 1 to 2 times/quarter ④ 1 to 2 times/year ⑤ No

16. In the past 12 months, have you ever gone fishing (or shrimp/snail fishing) in open water alone?

① 3 times or more/month ② 1 to 2 times/month ③ 1 to 2 times/quarter ④ 1 to 2 times/year ⑤ No

17. In the past 12 months, did you play with your peers around the pond (other wild water, swimming pool)?

① 3 times or more/month ② 1 to 2 times/month ③ 1 to 2 times/quarter ④ 1 to 2 times/year ⑤ No

18. In the past 12 months, have you ever dived or jumped in open water of

unknown depth?

① 3 or more times/month ② 1 to 2 times/month ③ 1 to 2 times/quarter ④ 1 to 2 times/year ⑤ No

19. In the past 12 months, have you ever drowned?

①No ②1 time ③2 times ④3 times ⑤4 times ⑥≥5 times

20. Do you remember the cause of your most recent drowning?

① Swimming ② Falling into the water ③ Saving someone ④ Diving or scuba diving

⑤ Attack of illness ⑥ Boat ride ⑦ Don't remember ⑧ No drowning

21. Do you remember this drowning happened in the month of

22．Do you remember the exact time of the drowning?

① Morning ② Noon ③ Afternoon ④ Evening ⑤ Don't remember ⑥ No drowning

23. Did anyone see the drowning?

① Yes ② No ③ Don't know ④ No drowning

24. Where was the drowning place?

① Bathtub ② Swimming pool ③ River/pond/reservoir ④ Well ⑤ Drain/puddle ⑥ Cesspool ⑦ Ditch/sewer ⑧ Lime pond ⑨ Don't know ⑩ No drowning

25. Did you save yourself or others after this drowning?

① Self-rescue - get to shore by yourself

② Other rescue - someone else rescued you

③ Don't know

④ Did not drown

26. When you were rescued, did you feel conscious?

① Yes ② No ③ Don't know ④ Did not drown

27. What did you do after drowning? (Fill in the most important treatment)

① No treatment ② Emergency treatment at the scene ③ Hospital outpatient (emergency) treatment ④ Hospitalization ⑤ Other ⑥ No drowning

28. What was the cost of the drowning treatment?

① No cost ② <10￥ ③ 10-99￥ ④ 100-999￥ ⑤ 1000-2999￥ ⑥ 3000-9999￥ ⑦ 10000-29999￥ ⑧ ≥30000￥ ⑨ Don't know ⑩ No drowning

29. In which area did you suffer a loss of function because of this drowning?

① No loss of function ② No use of hands or arms ③ Difficulty using hands or arms ④ Walking on cramped feet ⑤ Loss of hearing ⑥ Inability to chew food ⑦ Other ⑧ Refused to answer ⑨ Did not drown

Date of survey: MM/YY

This is the end of the form, please check again for any errors or omissions.

Thank you for your cooperation!

Table S1. Definitions and values of variables

variables	Definitions and values
Occurrence of drowning (y)*	No = 0, Yes = 1
Age (Years)	A1b<=9=1,10≤A1b≤14=2, 15≤A1b≤18=3.
Grade	Continuous variable (From 3 to 8)
Gender	Males = 1, Females = 2
Personality	Introvert = 1, Extrovert = 2, Between introvert and extrovert = 3, Do not know = 4
Relationships with classmates	Very good = 1, Good = 2, Not good = 3, Bad = 4
Relationships with family	Very good = 1, Good = 2, Not good = 3, Bad = 4
Number of siblings	One =1, Two = 2, Three =3, Four or over=4
Home ranking	Frist =1, Second =2, Third=3, Do not know = 9
If there are hazard warning signs on	Yes=1, Probably=2, Probably not=3,

ponds (or rivers, lakes, reservoirs, etc.), would you still play or swim there?	No=4
Distance from school to open water (Meters)	< 100 = 1, 100–500 = 2, 500 + =3, Have no water area = 4, Do not know = 5
Distance from home to open water (Meters)	< 100 = 1, 100–500 = 2, 500 + =3, Have no water area = 4, Do not know = 5
Are there open water areas on the way to school	Yes = 1, No = 2, Do not know = 3
Without the company of an adult, if a friend or classmate asks you to play or swim by the water, will you go?	Yes=1, Probably=2, Probably not=3, No=4
Swimming level (Meters)	≧100 = 1, 50–100 = 2, < 50 = 3, Unable to swim =4
In the past 12 months, have you ever been swimming in open water without the company of an adult?	More than three times a month=1, Once or twice a month=2, Once or twice a season=3, Once or twice a year=4, No=5
Whether to fish alone	More than three times a month=1, Once or twice a month=2, Once or twice a season=3, Once or twice a year=4, No=5

Playing around the pond	More than three times a month=1, Once or twice a month=2, Once or twice a season=3, Once or twice a year=4, No=5
Diving in waters of unknown depth	More than three times a month=1, Once or twice a month=2, Once or twice a season=3, Once or twice a year=4, No=5

* Occurrence of drowning: once had the experience of drowning=Yes, had on experience of drowning=No.
